# Draft Genome Sequences of 11 Clinical Isolates of *Acinetobacter baumannii*

**DOI:** 10.1128/genomeA.00269-16

**Published:** 2016-04-14

**Authors:** P. G. Higgins, J. Z.-M. Chan, H. Seifert, M. J. Pallen, A. D. Millard

**Affiliations:** a Institute for Medical Microbiology, Immunology and Hygiene, University of Cologne, Cologne, Germany; b German Center for Infection Research (DZIF), partner site Bonn-Cologne, Cologne, Germany; c Microbiology and Infection Unit, Warwick Medical School, University of Warwick, Warwick, United Kingdom

## Abstract

The development of multidrug-resistant *Acinetobacter baumannii* is of serious concern in the hospital setting. Here, we report draft genome sequences of 11 *A. baumannii* isolates that were isolated from a single patient over a 65-day period, during which time the isolates exhibited increased antimicrobial resistance.

## GENOME ANNOUNCEMENT

*Acinetobacter baumannii* is recognized as a serious threat in the hospital environment, particularly in the intensive care unit (ICU) setting (1). The treatment of *A. baumannii* infections can be complicated, because the organism is often multidrug resistant (MDR), mediated through acquired resistance determinants, or a mutant (2). In this study, we present the draft genome sequences of 11 MDR *A. baumannii* isolates that were isolated from a single patient over a 65-day period. Over this time period, the patient was treated with multiple antibiotics, a practice that is associated with increased antimicrobial resistance (3). This was reported previously to be associated with mutations in the genes encoding the efflux pump regulator AdeR and the acquired oxacillinase OXA-164 (3).

Bacterial genomic DNA was prepared from cultures using the Qiagen DNeasy kit, according to the manufacturer’s instructions, and sequenced using MiSeq (Illumina, USA). One nanogram of genomic DNA was prepared using the Nextera XT DNA sample preparation kit (Illumina) prior to sequencing on the MiSeq platform using the paired-end 2 × 250-bp (version 2) protocol. The resulting FASTQ files were quality trimmed and assembled *de novo* using the Velvet integrated in the Ridom SeqSphere+ version 3.0 software (Ridom GmbH, Münster, Germany). Here, reads were trimmed at their 5′ and 3′ ends until an average base quality of 30 was reached in a window of 20 bases, and the assembly was performed with Velvet version 1.1.04 using optimized k-mer size and coverage cutoff values based on the average length of contigs with >1,000 bp. All genomes were sequenced to a minimum depth of 20×, with a median coverage of 41× across all 11 isolates. The median genome size was 3.980 Mb and ranged from 3.878 Mb (isolate C) to 3.996 Mb (isolate K). The resulting number of contigs per genome ranged from 122 (isolate J) to 836 (isolate C). Contigs were annotated with Prokka 1.11 (4) using the *Acinetobacter*-specific database for annotation. The number of predicted tRNAs per genome ranged from 39 (isolate C) to 58 (isolate F). The range of predicted coding sequences (CDSs) per genome was from 3,543 CDSs (isolate C) to 3,797 CDSs (isolate I), with a median of 3,780 CDSs from all 11 genomes.

Multilocus sequence typing using the Pasteur scheme (http://pubmlst.org/abaumannii/) shows that all isolates belong to sequence type 1 (ST1), which corresponds to the international clone lineage 1 (5). The genome sequences confirmed the previously observed resistance-associated mutations (3). Furthermore, *in silico* analysis using the CGE server (https://cge.cbs.dtu.dk/) revealed that these isolates possess multiple aminoglycoside-modifying enzymes: all possessed *aac(3)-Ia* and *aph(3′)-Ic*, while nine amikacin-resistant isolates had *aph(3′)-via*, which was missing in two amikacin-susceptible isolates.

The genome information of these 11 sequential *A. baumannii* isolates will aid our understanding of genetic changes that occur during a prolonged infection treated with multiple antimicrobial therapies.

### Nucleotide sequence accession numbers.

The draft genome sequences of isolates B, C, D, E, F, G, I, J, K, L, and M have been deposited in DDBJ/ENA/GenBank under the accession numbers FITR01000001, FCNC01000001, FCND01000001, FCNG01000001, FCNJ01000001, FCNF01000001, FCNK01000001, FCNH01000001, FCNI01000001, FCNE01000001, and FJMY01000001, respectively.
